# 194 Evaluating the Acute Physiological Response of People Living with Dementia and Care Partners to a Ceili Dance Exercise Class

**DOI:** 10.1093/eurpub/ckae114.165

**Published:** 2024-09-26

**Authors:** Niamh Kelly, Fiona Skelly, Kieran Dowd, Desmond O’Neill, Noel McCaffery, Clare McDermott

**Affiliations:** Technological University Of The Shannon, Athlone, Ireland; Technological University Of The Shannon, Athlone, Ireland; Technological University Of The Shannon, Athlone, Ireland; Trinity College Dublin, Ireland; Tallaght University Hospital, Ireland; ExWell Medical, Ireland; Technological University Of The Shannon, Athlone, Ireland

## Abstract

Exercise prescription is designed using the FITT principle, this includes Frequency, Intensity, Time and Type 1. WHO recommends adults engage in 150 minutes of moderate exercise per week 2. It is difficult to verify the best methodology for reliable and validated results in exercise intensity 3. Exercise programs often do not know the intensity level of the delivered exercise. Research is further limited for people living with dementia (PLWD) and care partners 4. Determining exercise intensity is essential for understanding the most beneficial exercise for PLWD and care partners 4.

Participants (N = 15) attending a Ceili dance exercise class were recruited to take part in this study. Three variables to measure intensity were recorded for each participant during the class. Heart rate (HR) using a polar H10 monitor, rate of perceived exertion (RPE) using the modified Borg CR-10 scale and physical activity intensity using an ActivPAL monitor. Resting HR was recorded, on commencement of the class HR was documented every 5 minutes and a further 15 minutes after class. RPE was recorded after each section of the class. The ActivPAL recorded 15 second epochs throughout the class. All measures were analysed. HR and ActivPAL data were computed to determine intensity for each participant. The Karvonen formula was used to calculate HR intensity. SPSS version 25 will be used to statistically analyse the data.

There are currently preliminary results available for this study. These results are based on 3 participants. The average resting HR was 86bpm. The average HR for the warmup was 117bpm, dance section was 116bpm, strength section was 108bpm and cool down was 97bpm. Moderate intensity based on HR was found to be between 113 - 125bpm for this group. The average RPE for the class was 3. The ActivPAL data has yet to be analysed.

The results show the participants are working at moderate intensity during the main phase of the class. This confirms that dance exercise is an effective approach for PLWD to exercise at a moderate intensity.

This study is funded by the President’s Doctoral Scholarship Award from the Technological University of the Shannon.

